# Depression Is Associated with a Higher Risk of Mortality among Breast Cancer Survivors: Results from the National Health and Nutrition Examination Survey–National Death Index Linked Study

**DOI:** 10.3390/brainsci14070732

**Published:** 2024-07-21

**Authors:** Jagdish Khubchandani, Srikanta Banerjee, Kavita Batra, May A. Beydoun

**Affiliations:** 1 College of Health, Education and Social Transformation, New Mexico State University, Las Cruces, NM 88003, USA; 2 College of Health Sciences, Walden University, Minneapolis, MN 55401, USA; srikanta.banerjee2@mail.waldenu.edu; 3 Department of Medical Education, University of Nevada, Las Vegas, NV 89102, USA; kavita.batra@unlv.edu; 4 Laboratory of Epidemiology and Population Sciences, National Institute on Aging (National Institutes of Health), Baltimore, MD 21224, USA; baydounm@mail.nih.gov

**Keywords:** mortality, malignancy, depression, breast cancer, prevention

## Abstract

Breast cancer (BC) and depression are globally prevalent problems. Numerous reviews have indicated the high prevalence of depression among BC survivors. However, the long-term impact of depression on survival among BC survivors has not been well explored. For this investigation, we aimed to explore the relationship between BC, depression, and mortality from a national random sample of adult American women. Data from the U.S. National Health and Nutrition Examination Survey (years 2005–2010) were linked with mortality data from the National Death Index up to December 31st, 2019. A total of 4719 adult women (ages 45 years and older) were included in the study sample with 5.1% having breast cancer and more than a tenth (12.7%) having depression. The adjusted hazard ratio (HR) for all-cause mortality risk among those with BC was 1.50 (95% CI = 1.05–2.13) compared to those without BC. In the adjusted analysis, the risk of all-cause mortality was highest among women with both depression and BC (HR = 3.04; 95% CI = 1.15–8.05) compared to those without BC or depression. The relationship between BC and mortality was moderated by cardiovascular diseases, anemia, smoking, age, PIR, and marital status. Our analysis provides vital information on factors that could be helpful for interventions to reduce mortality risk among those with BC and depression. In addition, given the higher risk of mortality with co-occurring BC and depression, collaborative healthcare practices should help with widespread screening for and treatment of depression among BC survivors.

## 1. Introduction

Breast cancer (BC) is among the most frequently diagnosed and common cancers among women globally. Additionally, BC is the leading cause of cancer-related mortality in women worldwide [1,2]. According to recent statistics, more than 2 million women are diagnosed and more than half a million die annually of BC. It is estimated that these numbers will rise to more than 3 million new BC cases and more than a million BC deaths per year by the year 2040 [1,2,3]. Along with colorectal cancer deaths, BC deaths are the leading treatable causes of premature cancer deaths [2,3,4]. Despite advances in treatment and increasing awareness, BC deaths continue to escalate worldwide. This is in part due to the greater screening and survival in high-income countries and lower rates of prevention and treatment in low-income countries. Similarly, disparities in BC mortality also occur due to regional sociodemographic differences and access to healthcare services [3,4,5]. 

Within the past few decades, the role of comorbidities in predicting mortality among BC patients has also been explored [6,7,8,9]. An early review from 2013 suggested that more than a fifth of BC survivors may have comorbidities and in cohort studies with more than 5 years of follow-up, comorbidities led to a 1.1–5.8 times higher risk of mortality among BC survivors [6]. Recent studies have also suggested that the most common comorbidities that lead to decreased survival among BC patients include cardiovascular and metabolic diseases, chronic kidney diseases, and chronic obstructive pulmonary diseases [6,7,8,9]. However, the evidence is scattered about the role of comorbidities in predicting survival in BC patients given the variations in study samples globally (e.g., based on BC stage, treatments, sociodemographic factors, and the type of mortality assessed, etc.) [8,9,10,11,12].

Mental illnesses are a major group of comorbidities examined among BC survivors with depression receiving extensive attention in the published literature [12,13,14,15,16]. In a 2018 summary of the collective evidence, 33 out of the 38 included studies found higher rates of depression among BC survivors (with 19 reporting statistically significantly higher prevalence of depression) [13]. A review from 2019 found that among 72 studies performed in 30 countries, the prevalence of depression was 32.2% among BC survivors [14]. More recently, a meta-analysis from 2023 that included 71 studies from 26 countries found that the prevalence of depression in women with BC was 30.2% [15]. While these and other reviews have consistently confirmed a higher prevalence of depression among a variety of BC survivors (e.g., based on the stage of cancer and treatments received), the evidence regarding mortality risk among BC survivors with depression remains ambiguous. For instance, a study using Women’s Health Initiative data on more than 3000 women found no significant association between baseline depression and mortality among women with invasive BC [16]. Similarly, a study by Iglay and colleagues using SEER-Medicare data on 19,028 women aged 68 years or older found no association between depression and BC mortality among women diagnosed with stage I to IIIa BC [17]. In contrast, a study by Desai and colleagues using SEER-Medicare data among 10,452 BC survivors, who began adjuvant endocrine treatments, found that BC survivors with depression had a higher risk of mortality (HR = 1.44; 95% CI = 1.05–1.98) compared to those without depression [18]. Another study with data from the Korean National Health Insurance Service (2007–2014) on 124,381 BC patients assessed mortality among those who were diagnosed with depression after BC. The authors found that BC survivors with depression had a higher risk of mortality (HR = 1.26; 95% CI = 1.18–1.36) compared to those without depression [19]. Multiple other studies have shown equivocal findings [16,17,20,21].

The aforementioned studies assessing the association of depression with the risk of mortality among BC survivors had some limitations [15,16,17,18,19,20,21]. First, the studies were often limited by sample size or included older adults only. Second, these studies did not always account for a variety of sociodemographic characteristics (e.g., marital status or education). Third, many of these studies only investigated BC mortality, which may undermine the risk of other types of mortality among BC survivors with depression. Fourth, some of these studies did not account for major comorbidities observed among BC survivors (e.g., heart disease). Fifth, some of these studies had short follow-up durations to assess mortality among BC survivors with depression [16,17,20,21]. Therefore, we conducted a population-based data analysis to overcome some of these limitations by assessing the risk of mortality among women with BC or depression by utilizing a larger community-based sample of women, a relatively longer follow-up duration, and by accounting for numerous comorbidities and sociodemographic variables.

## 2. Methods

### 2.1. Study Sample and Measures

Data from three cycles of the National Health and Nutrition Examination Survey (NHANES, 2005–2006, 2007–2008, and 2009–2010) were analyzed in this secondary data analysis study by merging individual cycle data from the NCHS which provides the public use data on a biannual basis [19,20]. The NHANES has a complex, multi-stage, probabilistic sample design and includes participants who are representative of noninstitutionalized adult Americans. The National Death Index (NDI) files from the NCHS have publicly available mortality data for individuals via death certificates. The identifying information of all NHANES participants aged 18 and above (e.g., name, gender, date of birth, etc.) is matched between the NHANES and NDI databases. We linked deidentified mortality data collated by the NCHS with the survey participant data using probabilistic matching between NHANES and NDI records up to 31 December 2019. The cause of mortality was determined following the International Statistical Classification of Diseases and Related Health Problems, Tenth Revision (ICD-10). The follow-up period was determined by measuring time from the date of the survey interview and death or the end date of the study review (i.e., 31 December 2019) [22,23,24,25]. Before the NHANES data collection began, the U.S. Centers for Disease Control and Prevention’s NCHS Research Ethics Review approved all protocols and procedures (Protocol# 2005-2006).

The study participants were asked during the NHANES data collection “Has a doctor or other health professional ever told you that you had cancer or malignancy?”. Participants who answered “yes” were further asked” “Which kind of cancer was it?”. Those who selected “breast cancer” were considered to have the disease. Depression prevalence was evaluated by using the PHQ-9 survey data included in the NHANES database (collected via personal interviews of study participants) [26,27,28]. This extensively validated and reliable measure has nine questions with total scores ranging from 0 to 27 (scores 1–4 = minimal depression; 5–9 = mild depression; 10–14 = moderate depression; 15–27 = moderately severe or severe depression). For our analysis, this variable was dichotomized with individuals having a score ≥ 10 considered to have clinically relevant depression (CRD). When compared to an independent structured mental health professional interview, a PHQ-9 score ≥ 10 produces greater than 85% sensitivity and specificity for diagnosing CRD. Cohort studies frequently employ CRD symptoms as a proxy for depression [26,27,28].

We also considered study participant variables available in the NHANES database such as age groups, racial/ethnic background, marital status, educational attainment, health insurance availability, family income to poverty ratio (FIPR), current smoking status, and body mass index (BMI). Marital status was dichotomized into two categories for regression analyses: married and living with a spouse versus widowed, divorced, separated, and never married. Anemia was analyzed using self-reported data, as was cardiovascular disease (CVD) based on self-reported diagnoses of any one of the options affirmed by the study participants (i.e., coronary heart disease, angina, stroke, congestive heart failure, or heart attack). Chronic kidney disease (CKD) prevalence was determined using the standard Cockcroft–Gault equation-related data. Details about the NHANES and NDI data and analysis have been published extensively in multiple other studies [22,23,24,25,26,27]. 

### 2.2. Statistical Analysis

To characterize the study participants’ composition, their health and sociodemographic traits were analyzed using descriptive statistics (e.g., frequencies, percentages, and 95% confidence intervals). Next, group differences in health and sociodemographic characteristics were assessed between those with and without BC and those with and without clinically relevant depression by using Rao Scott chi-square tests (Table 1). Finally, multiple Cox regression models were constructed to examine differences in mortality among study participants based on whether or not they had BC or clinically relevant depression after adjusting for health-related and sociodemographic characteristics (Table 2). Those without BC or clinically relevant depression were used as a reference group (and compared to participants with BC only, depression only, or both BC and depression). Mortality was assessed by computing hazard ratios (HR, with 95% confidence intervals). These hazard ratios are adjusted for demographic characteristics (e.g., age, education, race/ethnicity, marital status), socioeconomic characteristics (e.g., health insurance, family poverty income ratio), behavioral characteristics (e.g., smoking), anthropometric (body mass index), and health status (e.g., comorbidities). Probability sampling weights considering NHANES nonresponse, oversampling, poststratification, and sampling errors were applied to improve the generalizability of the study sample at the individual participant level. All variance calculations accounted for the complex sample design using Taylor series linearization. Statistical significance was considered a priori at a level of *p* < 0.05. Statistical analysis was performed using SAS v.9.4 software.

## 3. Results

The NHANES 2005–2010 database had 31,034 participants, but we excluded males and those under the age of 45 years and included only those women for whom BC data (yes or no BC) were available. A final pool of 4719 adult females across the U.S. were included with 5.1% having breast cancer and more than a tenth (12.7%) having clinically relevant depression. Compared to women without BC, the women with BC were significantly more likely to be older, non-Hispanic White, widowed, and non-smokers. In addition, they were more likely to have normal weight, health insurance, college education, CKD, or CVD. (Table 1). In contrast, compared to those without depression, women with depression were significantly more likely to be smokers, non-White, non-married, obese, in poverty, younger (in the 45–54 year age group), and with a history of CVD. They were also less likely to have a college education, health insurance, or CKD.

Among women with BC, there were 35.2% deaths compared to 19.8% deaths among those without BC upon a mean of 7.6 years follow-up (Table 1). With regards to the risk of all-cause mortality, the unadjusted hazard ratio (HR) for those with BC was 2.26 (95% CI = 1.63–3.14, not shown in tables) compared to those who did not have BC. In the total population, irrespective of depression symptoms, the adjusted HR for all-cause mortality risk among those with BC was 1.50 (95% CI = 1.05–2.13) compared to those without BC (Table 2, column 2). In the stratified adjusted analysis (Table 2, column 5), the risk of all-cause mortality was significantly elevated among those with clinically relevant depression and BC (HR = 3.04; 95% CI = 1.16–8.01) compared to those who did not have BC and depression. However, for those with BC only and no depression (Table 2, column 3), the risk of mortality was not significantly higher (HR = 1.45, 95% CI = 0.99–2.12). Among women with clinically relevant depression but no BC (Table 2, column 3), the adjusted risk of all-cause mortality was also significantly higher (HR = 1.43, 95% CI = 1.09–1.86) compared to those without BC and depression. In these stratified analyses, CVD, CKD, anemia, obesity, smoking, age, PIR, ethnicity, and marital status were factors that predicted the relationship between BC, clinically relevant depression, and mortality across various comparison groups. The only factors that consistently moderated the relationship between BC and mortality across all comparison groups were CVD, anemia, smoking, age, PIR, and marital status providing vital information on factors that could be helpful for interventions to reduce mortality risk among those with BC and depression.

## 4. Discussion

Our analysis suggests that the risk of premature mortality among those with BC and depression is significantly elevated. It almost appears that depression and BC have an additive effect on reduction in survival as the risk of mortality from BC or depression alone if added together is lesser than the mortality rates among those with both depression and BC. These findings can be explained by a complex interplay of numerous factors along multiple psychosocial and biological pathways [16,18,20,21,29,30,31,32]. For psychosocial pathways, it is known that individuals with psychological distress may not be able to take care of themselves (e.g., unhealthy lifestyles), engage in health risk behaviors (e.g., substance use), might lack social and emotional support, or may not have the resources to adequately manage their cancer diagnosis and co-occurring health problems. Multimorbidity in individuals with CRD could impact their overall well-being and also result in poorer health outcomes. These factors could in turn result in the recurrence or progression of BC, inadequate treatment for BC, and premature mortality. For biological pathways, inflammation, and neuroendocrine or vascular disruptions may explain the higher mortality among BC survivors with depression. As an example, both cancer and depression have been related to inflammation, and the co-occurrence of these two disorders may increase systemic inflammation with the release of cytokines, increase procoagulant states and platelet aggregation, and endothelial dysfunction. Further evidence of how these pathways explain higher mortality among BC survivors with depression could be noted from the major causes of death among BC patients (e.g., cancers, heart disease, infections, and liver or kidney disease) that have been linked with aging, inflammation, presence of comorbidities, unhealthy lifestyles, and other biobehavioral factors [7,8,9,10,11,12].

The findings of this study have major public health practice implications. As noted earlier from major recent reviews, globally, more than a quarter of the women with BC may have depression and a third or more may have any mental illness [13,14,15]. Regular screening for psychological distress and adequate management of such disorders is warranted among BC survivors and can be accomplished by collaborative care models that engage oncologists and surgeons with psychiatrists and counselors. As our analysis found that age, income, and marital status play a major role in the relationship between BC, depression, and mortality, these interdisciplinary teams should also consider working with social workers to help BC survivors with their socioeconomic and emotional needs [9,10,11,12,32,33,34,35]. Health educators (e.g., dietitians) should also be consulted regularly to help improve and manage the lifestyles of BC survivors (our analysis indicates that more than a quarter of them are either overweight or have anemia and more than a tenth were current smokers). Specialists and primary care practitioners should assist in regular and aggressive management of comorbidities and risk behaviors among BC survivors given the high prevalence of comorbidities that may increase the risk of premature mortality. We found that CVD, CKD, anemia, obesity, and smoking are influential factors in predicting mortality risk among those with both BC and depression. Clinicians should be sensitized to such multimorbidity and unhealthy lifestyle behaviors among BC survivors so that they can diagnose, treat, or refer BC survivors for timely management, quality medical care, and lifestyle interventions to reduce premature mortality and improve the quality of life among those with BC [32,33,34,35].

The results of this analysis have several potential limitations [23,24,25,26,27]. Although cohort research designs are widely regarded as the most reliable in epidemiological literature, it is to be noted that observational studies cannot definitively demonstrate cause-and-effect relationships. Specifically, the database does not allow the assessment of temporal sequences (i.e., if breast cancer was diagnosed before the occurrence of depression symptoms or vice versa). Also, while we considered a broad sample of BC survivors with a longer follow-up duration, the sample sizes might have been insufficient to identify any interactions among the variables of interest, or longer durations of follow-up could have altered the results. While we used a national dataset of a random sample of community members, the lack of representativeness of our sample compared to those who did not participate in the NHANES could limit the external validity of our findings. Furthermore, the reliance on self-reported data from the NHANES could limit the validity of our findings (e.g., due to recall bias). Also, while we used the highly valid and reliable PHQ-9 scale to ascertain clinically relevant depression, using clinically diagnosed depression measures may have altered our findings. Finally, while we included numerous factors in the multivariable models, residual confounding cannot be ruled out and could potentially account for the observed associations (e.g., the influence of other lifestyle factors such as alcohol consumption, medications taken by the study participants, or other health conditions). Despite these restrictions, the major strengths of this study lie in the utilization of data from a diverse sample of adult women encompassing a wide age range of demographic groups in the U.S. Furthermore, this is one of the few investigations assessing BC, depression, and mortality in a population of BC survivors residing in the community. The linkage to NDI data until December 2019 provided sufficient statistical power to examine our hypotheses. We employed sophisticated statistical methods to conduct our analyses and adjusted for numerous potentially influential variables in the relationship between BC, mental health, and mortality.

Our findings and limitations of this study also suggest major implications and directions for future research [36,37,38,39,40,41,42,43,44]. First, well-designed prospective studies are needed to understand the relationship between BC, depression, and survival to ascertain temporality (e.g., studies where depression onset occurs before BC and vice versa). Second, multiple lifestyle factors (e.g., diet, substance use, religiosity), social factors (e.g., availability of social and medical support), and genetic and family history (e.g., medical history, familial risk, trauma, and adversity) should be measured in these prospective studies to understand their relative contribution in increasing or reducing survival among those with BC with or without depression. Third, given that a person with severe depression or BC might use some form of medical assistance (e.g., antidepressant medication or cancer therapies), the impact of such medical or complementary and alternative therapies should be assessed as it relates to survival among those with BC (e.g., based on our findings, it could be possible that timely therapy for depression might reduce the risk of mortality). Similarly, certain modifiers of depression treatment effects such as menopausal status or the use of adjuvant chemotherapy or hormone therapy should be thoroughly investigated among BC survivors. Fourth, BC survivors or those with depression are not a monolithic group (e.g., those with varying severity of depression or with different stages of breast cancer); the influence of BC and depression phenotype on mortality should be investigated. Additional subgroup investigations (e.g., based on age, race, or comorbid conditions) could also help with tailoring therapy and management of those with BC and depression. Fifth, depression frequently cooccurs with other physical and mental health problems among those with and without BC (e.g. anxiety). The influence of such psychological and physical multimorbidity should be investigated for their impact on BC occurrence, remissions, and mortality by cause. Sixth, and finally, there is a need for research on personalized approaches to improving psychological health among BC survivors. Such an approach should consider individual characteristics, social and contextual factors, psychiatric and psychological history, psychological and emotional response to BC diagnosis, BC profile and cancer characteristics, and treatments preferred or received for BC and depression [36,37,38,39,40,41,42,43,44]. Given the various profiles of BC survivors, the widely wavering and complicated nature of depressive symptoms and contributing medical, sociodemographic, and psychosocial factors, these research priorities are key to establishing evidence-based guidelines for the use of pharmacotherapy and/or psychotherapy among BC survivors with mood disorders (e.g., depression).

## 5. Conclusions

This population-based nationwide study from the U.S. underscores the additive effect of BC and depression on the risk of mortality. As indicated by the findings, the absolute risk of mortality among BC survivors is relatively lower than among those with both BC and depression. These findings indicate the need for multifaceted approaches to develop comprehensive and well-coordinated survivorship care models for those with BC. The findings also highlight the need for an integrated approach to managing physical as well as psychological health among those with BC. Specifically, cancer treatment facilities should offer screening and counseling services for mental illnesses as a part of their patient treatment and management plans to maximize survival among BC survivors. Future studies should emphasize the exploration of psychological burdens beyond depression among BC survivors, specifically among high-risk and vulnerable groups. Research is also needed on rapid and cost-effective primary care-based interventions that can be widely deployed to improve the mental health of BC survivors.

## Figures and Tables

**Table 1:** Characteristics of Study Participants Stratified by Breast Cancer Diagnosis and Depression Symptoms.

Characteristics	Total Population (n = 4719)	Breast Cancer (+) (n = 240)	Breast Cancer (−) (n = 4479)	Depression + (n = 479)	Depression − (n = 2526)
**Age** (Mean, years)	60.8 (60.2–61.3)	66.3 (64.1–68.5) **	60.5 (59.9–61.0)	57.0 (55.7–58.2)	60.7 (60.0–61.3) *
**Age Group** (%, 95% CI)					
45–54 Years	38.0 (35.8–40.1)	17.4 (12.3–22.6)	38.8 (36.6–41.1) **	51.2 (44.3–58.1) **	37.7 (35.1–40.2)
55–64 Years	26.6 (24.9–28.5)	20.8 (15.1–26.6)	26.8 (25.1–28.5)	30.0 (24.3–35.7)	26.4 (24.3–28.5)
≥65 Years	35.5 (33.0–38.0)	61.7 (54.3–69.2)	34.3 (31.8–36.9)	18.8 (14.0–23.7)	35.9 (33.2–38.6)
**Family Poverty-Income-Ratio** (PIR < 1)	10.6 (9.3–11.9)	9.6 (6.0–14.9)	10.7 (9.3–12.0)	37.9 (31.1–44.7) **	21.2 (19.0–23.5)
**No Health Insurance** (%, 95% CI)	11.2 (9.6–12.9)	4.1 (1.9–8.8)	11.6 (9.9–13.4) **	23.2 (18.8–27.6) **	16.8 (14.8–18.8)
**Education Level** (%, 95% CI)					
Some High School	20.1 (18.2–22.12)	15.9 (10.0–21.8)	20.4 (18.3–22.4) *	42.3 (37.1–47.6) **	32.4 (29.2–35.5)
High School Graduate	25.3 (23.5–27.1)	23.1 (15.8–30.4)	25.4 (23.7–27.1)	23.0 (18.1–27.9)	25.1 (22.5–27.6)
Some College and Beyond	54.6 (51.6–57.6)	61.0 (52.2–69.8)	54.2 (51.3–57.1)	34.7 (29.0–40.3)	42.6 (39.7–45.4)
**Race/Ethnicity** (%, 95% CI)					
Non-Hispanic White	75.4 (71.8–79.1)	85.9 (81.5–90.3) **	74.6 (71.1–78.6)	36.5 (29.1–43.9)	44.8 (38.0–51.5) **
Non-Hispanic Black	11.0 (8.8–13.1)	8.2 (5.8–11.5)	11.1 (9.0–13.3)	30.3 (23.4–37.1)	29.8 (24.9–34.7)
Hispanic	8.3 (6.5–10.5)	3.0 (1.9–4.9)	8.6 (6.7–10.8)	30.5 (21.8–39.1)	22.4 (17.6–27.2)
Other	5.3 (4.3–6.6)	2.9 (1.0–7.7)	5.4 (4.4–6.8)	2.8 (1.3–5.8)	3.0 (2.1–4.4)
**Marital Status** (%, 95% CI)*/**					
Married	57.0 (54.7–59.2)	59.8 (52.3–67.3)	56.8 (54.5–59.1)	36.9 (31.8–41.9) **	45.4 (43.0–47.9)
Widowed	17.5 (16.1–18.9)	25.7 (20.7–30.8) **	17.0 (15.6–18.5)	17.4 (13.9–21.0)	21.6 (19.7–23.6)
Divorced	14.8 (13.1–16.5)	9.7 (6.3–14.8)	15.1 (13.4–16.9)	20.6 (16.3–24.9)	18.5 (16.4–20.6)
Separated	2.3 (1.8–2.8)	1.1 (0.5–2.8)	2.3 (1.9–2.9)	9.5 (6.4–14.0)	3.7 (2.8–4.9)
Never Married	5.6 (4.7–6.6)	1.4 (0.5–4.1)	5.8 (5.0–6.8)	10.0 (7.2–13.5)	8.1 (6.6–10.1)
Living with Partner	2.8 (2.3–3.6)	2.2 (0.6–7.7)	2.9 (2.3–3.6)	5.6 (3.6–8.7)	2.6 (2.0–3.4)
**Smoking** (current %, 95% CI)	15.9 (14.2–17.60	11.2 (8.5–15.9)	16.2 (14.4–17.9) *	35.5 (30.1–40.9) **	17.9 (15.6–20.3)
**Body Mass Index** (%, 95% CI)					
Normal Weight < 25	31.9 (30.3–33.5)	38.8 (30.4–47.2) *	31.5 (29.8–33.3)	19.3 (13.5–25.1) *	24.3 (22.4–26.1)
Overweight = 25–29.9	29.8 (27.9–31.6)	23.0 (17.9–29.0)	30.1 (28.1–32.1)	24.9 (19.0–30.9)	28.7 (26.9–30.5)
Obese = 30–39.9	30.1 (28.8–31.5)	29.5 (23.8–35.1)	30.2 (28.7–31.7)	37.7 (32.9–42.5)	35.9 (33.7–38.0)
Morbidly Obese = BMI ≥ 40	8.2 (7.3–9.2)	8.3 (4.3–15.4)	8.2 (7.3–9.1)	18.1 (14.2–22.0)	11.1 (9.4–12.9
**Anemia** (yes, %, 95% CI)	26.4 (23.7–29.0)	29.4 (22.7–36.2)	26.2 (23.4–29.0)	33.1 (27.2–39.1)	34.0 (30.9–37.2)
**Cardiovascular Disease** (%, 95% CI)	12.8 (11.4–14.2)	15.9 (10.4–21.5) *	12.6 (11.1–14.1)	24.4 (19.6–29.2) **	14.9 (13.1–16.7)
**Chronic Kidney Disease** (%, 95% CI)	18.8 (17.3–20.3)	36.4 (27.8–45.0) **	17.9 (16.5–19.3)	9.6 (6.6–13.8)	18.6 (16.2–21.0) **
**Depression** (%, 95% CI)	12.7 (11.5–13.9)	10.9 (4.5–17.3)	12.8 (11.5–14.1)	N/A	N/A
**All deaths** (N, %)	1159 (24.6)	104 (43.4) **	1055 (23.58)	117 (24.4)	594 (23.5)

Note. * *p* < 0.05 ** *p* < 0.01. Numbers with 95CI indicate 95% confidence intervals for proportions.

**Table 2:** Risk of Mortality Among Study Participants Based on Depression or Breast Cancer Diagnosis.

Predictors of Mortality	Total Population HR (95% CI) BC vs. No BC	Breast Cancer Only HR (95% CI)	Depression Only HR (95% CI)	Breast Cancer and Depression HR (95% CI)
**Comparison groups**	1.50 (1.05–2.13) *	1.45 (0.99–2.12)	1.43 (1.09–1.86) *	3.04 (1.16–8.01) **
**Cardiovascular Disease** (No = ref)	2.43 (2.04–2.91) **	2.42 (2.00–2.91) **	2.36 (1.92–2.90) **	2.01 (1.05–3.85) *
**CKD** (No = ref)	1.41 (1.03–1.92) *	1.34 (1.00–1.81)	1.51 (1.08–2.11) *	2.42 (1.01–5.79) *
**Anemia** (No = ref)	1.71 (1.40–2.09) **	1.63 (1.32–2.01) **	1.73 (1.38–2.16) **	2.19 (1.21–3.98) *
**Obesity** (No = ref)	1.30 (1.02–1.66) *	1.24 (0.94–1.62)	1.32 (1.03–1.69) *	2.34 (1.04–5.23) *
**Smoking Status** (No = ref)	1.57 (1.30–1.89) **	1.55 (1.23–1.97) **	1.55 (1.28–1.87) **	1.70 (1.01–2.92) *
**Age** (years)	1.09 (1.07–1.11) **	1.10 (1.08–1.12) **	1.09 (1.07–1.11) **	1.08 (1.05–1.12) **
**Family Poverty-Income-Ratio** (Ref: PIR ≥ 1)	1.53 (1.22–1.91) **	1.33 (1.03–1.71) *	1.51 (1.19–1.92) *	2.49 (1.32–4.68) *
**Health Insurance** (yes = ref)	1.25 (0.82–1.88)	1.24 (0.74–2.79)	1.20 (0.79–1.82)	0.97 (0.44–02.180)
**Education Level**				
Some College and Beyond	Ref	Ref	Ref	Ref
Some High School	1.29 (1.00–1.66)	1.33 (0.99–1.79)	1.36 (1.03–1.78)	0.78 (0.47–1.32)
High School Graduate	1.01 (0.84–1.28)	1.07 (0.86–1.34)	1.10 (0.87–1.38)	0.58 (0.29–1.16)
**Race/Ethnicity**				
Non-Hispanic White	Ref	Ref	Ref	Ref
Non-Hispanic Black	0.81 (0.63–1.05)	0.80 (0.60–1.07)	0.78 (0.59–1.04)	0.89 (0.38–2.08)
Hispanic	0.54 (0.34–0.84) *	0.60 (0.38–0.97) *	0.49 (0.33–0.74) *	0.34 (0.13–0.87) *
Other	0.56 (0.26–1.18)	0.45 (0.16–1.26)	0.54 (0.25–1.16)	0.77 (0.33–1.76)
**Marital Status** (ref = married/with partner)	1.44 (1.18–1.76) **	1.59 (1.28–1.97) *	1.34 (1.10–1.63) *	1.64 (1.44–1.94) *

Note. * *p* < 0.05 ** *p* < 0.01. HR (95CI) indicates hazard ratios with 95% confidence intervals for the outcome (i.e., mortality). Ref indicates the reference group among each variable for comparison with other groups.

## Data Availability

All the study data are publicly available at the U.S. Centers for Disease Control and Prevention NHANES website—https://www.cdc.gov/nchs/nhanes/index.htm (accessed 27 April 2024).
